# Developing an anticancer copper(II) pro-drug based on the nature of cancer cell and human serum albumin carrier IIA subdomain: mouse model of breast cancer

**DOI:** 10.18632/oncotarget.11465

**Published:** 2016-08-22

**Authors:** Yi Gou, Yao Zhang, Jinxu Qi, Shifang Chen, Zuping Zhou, Xiaoyang Wu, Hong Liang, Feng Yang

**Affiliations:** ^1^ State Key Laboratory for The Chemistry and Molecular Engineering of Medicinal Resources, Ministry of Science and Technology of China, Guangxi Normal University, Guilin, Guangxi, China; ^2^ Guangxi University Key Laboratory of Stem Cell and Pharmaceutical Biotechnology, Guangxi Normal University, Guilin, Guangxi, China; ^3^ Ben May Department for Cancer Research, University of Chicago, Chicago, IL, USA

**Keywords:** copper pro-drug, human serum albumin, tumor targeting, therapeutic effect

## Abstract

Human serum albumin (HSA)-based drug delivery systems are promising for improving delivery efficiency, anticancer activity and selectivity of anticancer agents. To rationally guide to design HSA carrier for anticancer metal agent, we built a breast mouse model on developing anti-cancer copper (Cu) pro-drug based on the nature of IIA subdomain of HSA carrier and cancer cells. Thus, we first synthesized a new Cu(II) compound derived from tridentate (*E*)-*N'*-(5-bromo-2-hydroxybenzylidene)benzohydrazide Schiff base ligand (HL) containing 2 potential leaving groups [indazole (Ind) and NO_3_^−^], namely, [Cu(L)(Ind)NO_3_]. Structural analysis of the HSA complex showed that Cu(L)(Ind)(NO_3_) could bind to the hydrophobic pocket of the HSA IIA subdomain. Lys199 and His242 coordinate with Cu^2+^ by replacing the indazole and NO_3_ ligands of [Cu(L)(Ind)NO_3_]. The release behavior of the Cu compound from the HSA complex is different at different pH levels. [Cu(L)(Ind)NO_3_] can enhance cytotoxicity by 2 times together with HSA specifically in cancer cells but has no such effect on normal cells *in vitro*. Importantly, our *in vivo* results showed that the HSA complex displayed increased selectivity and capacity to inhibit tumor growth and was less toxic than [Cu(L)(Ind)NO_3_] alone.

## INTRODUCTION

After the unexpected discovery of the antiproliferative activity of *cis*platin, many studies have highlighted potent metal-based drugs (metallodrugs) for treating cancers [1, 2]. Most anticancer metallodrugs have been studied both *in vitro* and *in vivo*, and some of these drugs have been tested in clinical trials [3–5]. Even though progress has been made to understand the chemistry of metallodrugs, these emerging therapeutics are associated with concerns such as a lack of water solubility and chemical deficiencies [3, 6–8]. To overcome these concerns, novel drug delivery vehicles must be developed [9–13].

Over the past several decades, various drug delivery systems, including pro-drugs, protein biomolecules, and nanoparticles, which maximize the efficacy of novel metallodrugs, have been developed [14–18]. Human serum albumin (HSA)-based delivery systems provide an interesting approach because HSA is a nontoxic, biocompatible and biodegradable endogenous protein that does not provoke an immune response and does not stimulate the production of antibodies [19]. HSA is the most abundant protein in blood plasma and contains a variety of special active residues, including cysteine and lysine [20]. The efficiency and selectivity of anticancer agents can be improved by chemically coupling these agents with the special residues of albumin [21–27]. However, this method often introduces exogenous chemicals in or changes the conformation of albumin [25–28]. HSA has 3 major binding sites for binding various exogenous compounds [29–32]. Thus, to overcome this, drugs can be complexed with HSA. Recent studies have shown that the specificity and efficacy of anticancer drugs can be enhanced by complexing them with HSA [33–37]. Interestingly, based on the nature of HSA IIA subdomain, Yang et al. proposed and built a model *in vitro* on developing copper anticancer drugs by pro-drug strategy [37].

**Figure 1:**
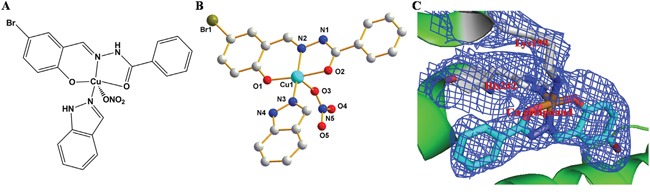
**A.** and **B.** Chemical structure of [Cu(L)(Ind)NO_3_]. **C.** The experimental *σ*A weighted 2*F*_o_ − *F*_c_ electron density map (blue, 1σ) of Cu pro-drug in HSA.

**Figure 2:**
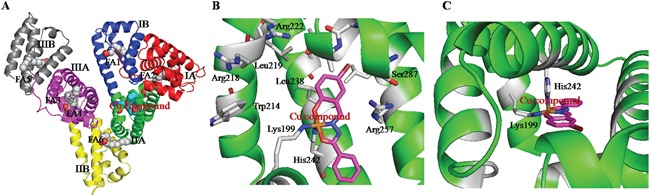
**A.** The overall structure of HSA complex. **B** and **C.** The structural binding environment of Cu prodrug in HSA from different angles.

Metallodrugs offer biological and chemical diversity that is distinct from that offered by organic drugs [38]. Biological activity, particularly antitumor activity, of various metals that form a complex with organic ligands is higher than that of just the organic molecules alone [39–41]. Cu-containing drugs may serve as promising next-generation anticancer agents because Cu is bioactive and serves as an essential element for human physiological functions [42, 43]. Breast cancer is the most prevalent cancer in women, and it is the leading cause of cancer deaths among women worldwide [44]. Although Yang et al. showed that a Cu pro-drug designed based on the nature of HSA IIA subdomain had enhanced selectivity and anticancer efficiency to some extent *in vitro*, its anticancer behavior *in vivo* was unclear [37]. Therefore, we used the pro-drug strategy to develop an HSA carrier for delivering the Cu compound *in vivo* for treatment of breast cancer by performing the following studies: (1) synthesizing a new aroylhydrazone Schiff base-derived Cu(II) compound containing the 2 leaving groups (Figure 1A), (2) confirming the feasibility of developing an anticancer Cu pro-drug exploiting the function of cancer cells and by using the HSA IIA subdomain, (3) determining whether the HSA carrier increased the selectivity and therapeutic efficacy of [Cu(L)(Ind)NO_3_] relative to that of [Cu(L)(Ind)NO_3_] alone *in vivo*, (4) determining whether the HSA complex was associated with less side effects than [Cu(L)(Ind)NO_3_] alone *in vivo*, and (5) determining the potent anticancer mechanism of [Cu(L)(Ind)NO_3_]/HSA complex.

## RESULTS

### Development and structure of the Cu-containing pro-drug

We developed a Cu-containing pro-drug with improved *in vivo* selectivity and drug delivery and anticancer efficiency using the structure of cancer cells as well as by using the HSA IIA subdomain. First, we used a tridentate (*E*)-*N'*-(5-bromo-2-hydroxybenzylidene) benzohydrazide Schiff base ligand (HL) for developing the Cu compound (pharmacophore) because the hydrazone class ligands are promising anticancer agent [45–47] and because its hydrophobic (log *P* = 3.8) and rigid structure may facilitate the binding of the Cu compound to the HSA IIA subdomain [37]. We selected NO_3_^−^ and indazole as the second and third ligands (potential leaving groups), respectively (Figure 1A). [Cu(L)(Ind)NO_3_] crystallizes in a triclinic system with a space group *P*−1. The asymmetric units contained 1 tridentate Schiff base ligand, 1 Cu(II) center, 1 indazole ligand, and 1 NO_3_^−^ (Figure 1B).

The Cu(II) metal center, which was formed by combining a nitrogen atom and two oxygen atoms from the Schiff base ligand, a nitrogen atom from the indazole ligand, and one terminal NO_3_^−^. The coordination of a polyhedron around the Cu1 center formed a distorted square pyramid (*τ =* 0.12) [48], with the metal displaced from the O1/N2/O2/N3 basal plane (maximum displacement of 0.09 Å for oxygen atom), and with NO_3_^−^ at the apex (metal displacement by 0.142 Å toward NO_3_^−^ from the mean basal plane). Cu−N and Cu−O bond distances were in the range of 1.879−2.474 Å, which were similar to those reported previously [37, 49–51]. Considerable strain existed in the coordination plane around the Cu(II) center because of the short bite (173.8°) of the O1−N2−O2 portion of the Schiff base ligand. The C7−O2 bond distance in the complex was shorter than that in the free ligands [52], which supported the formation of alkoxide after complexation. Dimers were formed in a solid state through N−H···O reactions, which involved a nitrogen atom (N1) from the Schiff base ligand and NO_3_^−^ (O3^i^) bonded to Cu1^i^ from an adjacent molecule (N1···O3^i^ = 2.902 Å; N1−H1···O3^i^ angle is 152.6°; symmetry code: (i) 1 − *x*, − *y*, 2 − *z*; Supplementary Figure S1). A small proportion of π···π stacking (Supplementary Figure S1) was observed between pyridine rings in [Cu(L)(Ind)NO_3_], which provided some stability to the dimeric structure.

### Feasibility of developing the HSA-based Cu pro-drug

The fluorescence of HSA at approximately 348 nm was gradually quenched by intensifying the concentration of [Cu(L)(Ind)NO_3_] (Supporting Information, Supplementary Figure S2A), suggesting that the Cu compound is bound closely to the IIA subdomain of HSA [53]. UV−Vis measurements suggested that the Cu compound can interact with HSA (Supplementary Figure S2B) [54]. Consistently, the MALDI-TOF-mass spectra indicates growth of about 400 Da for the HSA complex as compared to HSA alone, which was equivalent to the molecular weight of approximately one Cu compound [Cu(L)] (Supplementary Figure S2C).

To confirm whether the Cu prodrug could be developed using the HSA IIA subdomain, we determined the structure of the HSA−PA−[Cu(L)(Ind)NO_3_] complex. The electron density map of the Cu compound in the HSA complex clearly showed one Cu compound molecule in the IIA subdomain (Figures 1C and Supplementary Figure S3). Overall, the HSA−PA−[Cu(L)(Ind)NO_3_] complex was heart shaped (Figure 2A), which was comparable to the previously discovered structures of HSA−FA complexes [55], save for a slight structural change in the amount of adjacent chains at the drug-binding site. In the HSA IIA subdomain, the Cu compound was bound to a big hydrophobic pocket containing residues Arg218, Arg222, Lys199, Trp214, Leu219, Phe223, Leu234, Leu238, His242, Arg257, Leu260, Ile264, Ser287, Ile290, and Ala291 (Figure 2B and 2C). Of these residues, Lys199 and His242 coordinated with Cu^2+^ of [Cu(L)(Ind)NO_3_] by replacing indazole and NO_3_^−^, respectively (Figure 2B). Furthermore, the hydrophobic group of the Cu compound showed reactions with the side chains of surrounding residues. The nearest distances between the hydrophobic group and the adjacent side chains of HSA ranged from approximately 3.0 to 5.0 Å.

### *In vitro* anticancer activity of the HSA complex

The cytotoxicity of [Cu(L)(Ind)NO_3_] against MCF-7 cells (1.53 ± 0.14 μM) was higher than that of *cis*platin (18.34 ± 1.67 μM). The HSA complex showed approximately 2-fold higher toxicity against MCF-7 cells than [Cu(L)(Ind)NO_3_] alone but exerted less toxic effects on WI-38 normal lung fibroblast cells (Tables 1 and Supplementary Table S3). In addition, the [Cu(L)(Ind)NO_3_] compound and HSA complex exhibit no multidrug-resistant behaviors as exhibited by their skill in eliminating all doxorubicin-sensitive and -resistant MCF-7 and MCF-7/ADR cells (Table 1) with no apparent pattern.

**Table 1:** IC_50_^a^ (μM) values of (*E*)-*N'*-(5-bromo-2-hydroxybenzylidene)benzohydrazide Schiff base ligand (HL), Cu(II) compound and HSA complex toward a panel of human cell lines for 48 h

Compound	Antitumor activity IC_50_ (μM)		
	MCF-7	MCF-7/ADR	WI-38
HL	86.2 ± 6.5	89.5 ± 6.2	>100
[Cu(L)(Ind)NO_3_]	1.53 ± 0.14	1.78 ± 0.16	2.58 ± 0.23
HSA-[Cu(L)]	0.69 ± 0.08	0.92 ± 0.11	2.61 ± 0.24
*Cis*platin	18.34 ± 1.67	20.34 ± 1.82	18.21 ± 1.35
Doxorubicin	3.54 ± 0.29	>100	-

a IC_50_ values are presented as the mean ± SD from three separated experiments.

### *In vivo* studies of the HSA pro-drug

To evaluate whether the HSA complex showed enhanced therapeutic efficacy *in vivo*, we conducted the studies on the xenograft MCF-7 murine breast cancer model.

### Acute toxicity of the HSA complex and [Cu(L)(Ind)NO_3_]

Acute toxicities of the HSA complex and [Cu(L)(Ind)NO_3_] were assessed using normal mice. Serum parameters determined at 3 days after the intravenous administration of the HSA complex and Cu(L)(Ind)NO_3_ are listed in Table 2. [Cu(L)(Ind)NO_3_] induced significant nephrotoxicity, with its BUN value (20.3 ± 2.4 mmol/L) being higher than that of vehicle control (NaCl, 6.3 ± 1.1 mmol/L). While, the BUN (9.2 ± 1.3 mmol/L) of HSA complex is similar to NaCl group, indicating a lower nephrotoxicity caused by HSA complex than [Cu(L)(Ind)NO_3_]. Serum AST and ALT levels were significantly higher in [Cu(L)(Ind)NO_3_]-treated mice than in NaCl-treated mice. In contrast, i.v. administration of the HSA complex induced lesser changes in AST and ALT levels, suggesting that the HSA complex exerted lower hepatotoxicity than free [Cu(L)(Ind)NO_3_]. However, CK levels of HSA complex- and [Cu(L)(Ind)NO_3_]-treated mice were similar to those of control mice, indicating negligible cardiotoxicity.

**Table 2:** Serological analysis (creatinine kinase CK, blood urea nitrogen BUN, alanine aminotransferase ALT and aspartate aminotransferase AST) of mice injected NaCl, Cu(L)(Ind)NO3 and HSA-Cu(L)

Complex	CK (U/L)	BUN (mmol/L)	ALT (U/L)	AST (U/L)
NaCl	341 ± 13	6.3 ± 1.1	38.2 ± 8	89.3 ± 11
Cu(L)(Ind)NO_3_	382 ± 32	20.3 ± 2.4	71.3 ± 6.4	139 ± 15
HSA-Cu(L)	356 ± 26	9.2 ± 1.3	54.6 ± 5.7	109 ± 8.6

### Antitumor activity of the HSA complex and [Cu(L)(Ind)NO_3_]

**Figure 3:**
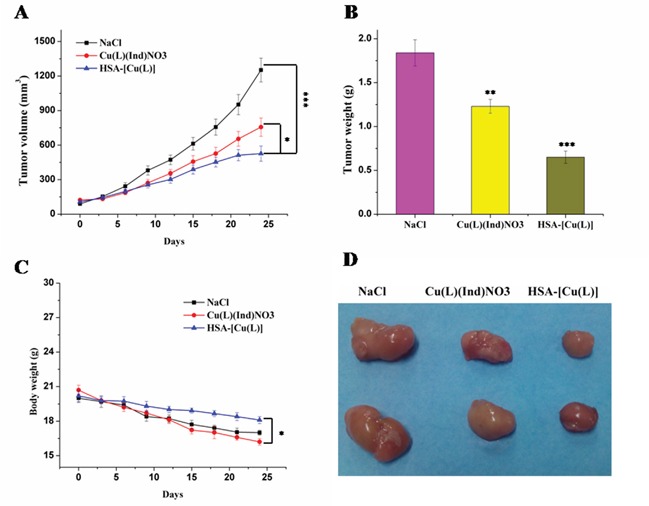
**A.** Variations of tumor volume after treatment with saline, [Cu(L)(Ind)NO_3_] and HSA-[Cu(L)]. **B.** Mean weight of tumors separated from mice after different treatments. **C.** Body weight changes of different formulations. **D.** Representative tumors separated from animals after intravenous injection of saline, [Cu(L)(Ind)NO_3_] and HSA-[Cu(L)]. Statistical significance: *p < 0.05; **p < 0.01; ***p < 0.001.

**Figure 4:**
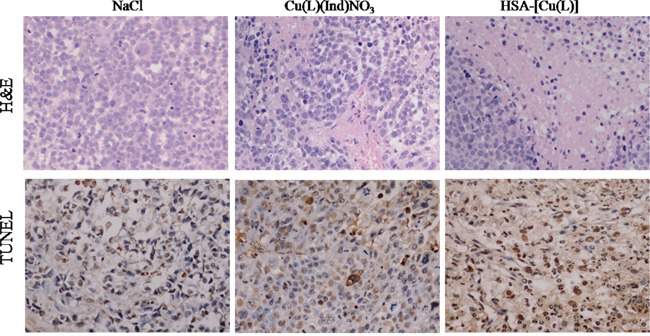
**A.** Tumors are sectioned and stained with H&E (magnification ×400). **B.** Apoptotic cells were detected in xenograft tumor tissue using the TUNEL assay.

The *in vivo* antitumor effect of the HSA complex and [Cu(L)(Ind)NO_3_] was evaluated using MCF-7 tumor-bearing mouse model. Variations in tumor volume and mouse body weight were monitored every 3 days for 24 days (Figure 3). At the end of this period, the tumors in nude mice receiving NaCl grew quickly, attaining an average net volume of 1252 ± 104 mm^3^. Compared with NaCl, the HSA complex and [Cu(L)(Ind)NO_3_] decreased the net volume of MCF-7 tumor xenografts after 24 days of treatment. Importantly, the HSA complex significantly decreased tumor volume compared with NaCl (*p* < 0.001; Figure 3A). The Tumor inhibitory rate (TIR) was computed using differences in tumor weight (Figure 3B). The TIR of the HSA complex was approximately 64.6 ± 8.7%, which is higher than that of [Cu(L)(Ind)NO_3_] alone (33.2 ± 5.2%). Histological examination with hematoxylin and eosin (H&E) staining and terminal deoxynucleotidyl transferase dUTP nick end labeling (TUNEL) assay were performed to investigate the tumor suppression efficiency of the HSA complex. Tumor cells of mice in the control group were normal and showed no visible apoptosis or necrosis (Figure 4). In contrast, different degrees of tumor cell apoptosis or necrosis were observed in mice treated with the HSA complex and [Cu(L)(Ind)NO_3_]. Mice treated with the HSA complex showed larger areas of apoptosis and necrosis than those treated with [Cu(L)(Ind)NO_3_], indicating that the *in vivo* antitumor efficacy of the HSA complex was superior to that of [Cu(L)(Ind)NO_3_].

### Comparison of the side effects of [Cu(L)(Ind)NO_3_] and the HSA complex

**Figure 5:**
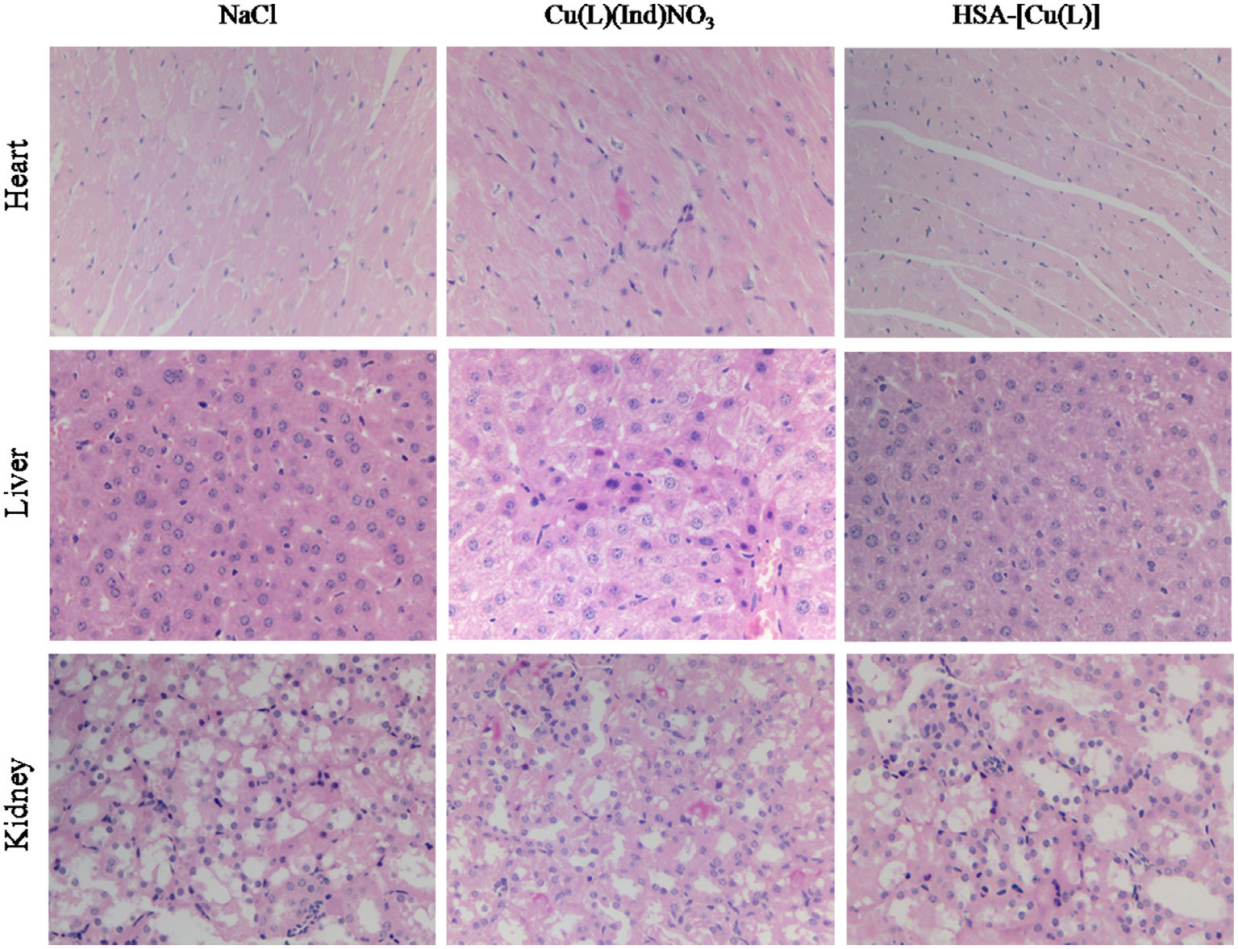
H&E staining analysis of organs sections treated with various treatments (magnification ×400)

Figure 3C shows the changes in the body of mice treated with NaCl, [Cu(L)(Ind)NO_3_], and HSA complex. Loss of body weight in mice treated with the HSA complex was only approximately 11.4 ± 0.9% from the original weight, which was lower than that observed in mice treated with [Cu(L)(Ind)NO_3_] (approximately 19.3% of the original weight), indicating that the HSA complex reduced the side effects induced by free [Cu(L)(Ind)NO_3_] (Figure 3C). In addition, drug-related side effects to major organs were observed by pathological section with H&E staining (Figure 5). Serious damage to the liver (hepatic cell atrophy, hepatic cord loss, and mild steatosis) and kidneys (focal abnormalities) was observed in mice treated with [Cu(L)(Ind)NO_3_]; this damage was greatly decreased in mice treated with the HSA complex. Interestingly, the hearts of mice in all the treatment groups showed normal histology.

### *In vivo* selectivity of the HSA complex

**Figure 6:**
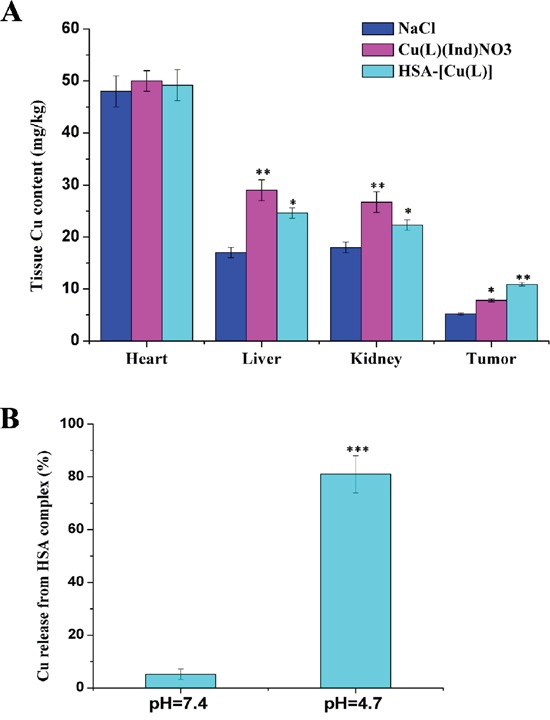
**A.** Tissue copper of mice after treatment with saline, [Cu(L)(Ind)NO_3_] and HSA-[Cu(L)]. **B.** The profiles of Cu release from HSA complex at different pH (citric-phosphate buffer). Results are the mean ± SD (n = 3): *p < 0.05; **p < 0.01; ***p < 0.001.

To evaluate whether the HSA complex selectively accumulated into MCF-7 tumor cells *in vivo*, we tested Cu concentration in the tumors of mice at the end of the experimental period. Results of inductively coupled plasma atomic emission spectrometry (ICP-AES) showed that Cu concentration in the MCF-7 tumors of mice treated with the HSA complex was approximately 1.5-fold higher than that in the tumors of mice treated with [Cu(L)(Ind)NO_3_] (Figure 6A). These data suggested that the HSA complex selectively accumulated in tumors. Furthermore, our data indicated that the HSA complex decreased Cu compound accumulation in the liver and kidney (Figure 6A).

### Release of Cu compound from the HSA complex

It remains unclear how the Cu compound is released from the HSA-[Cu(L)] complex under different environments. To address this issue, we simulated the *in vivo* environment and measured the release of the Cu compound from the HSA in buffers with pH 4.7 and 7.4 (Figure 6B) [56, 57]. Our results indicate that approximately 5% of the loaded Cu compound was released from the HSA complex within 48hours at pH 7.4 while up to 80% of the loaded Cu compound was released from the HSA complex at pH 4.7. Additionally, the binding affinity of [Cu(L)(Ind)NO_3_] to the HSA carrier at pH 7.4 (*K* = 7.03 ± 0.06 × 10^6^ M^−1^) was significantly stronger than that at pH 4.7 (*K* = 4.73 ± 0.03 × 10^4^ M^−1^). These results strongly suggest that the Cu compound was bound weakly to and could be released more easily from the HSA complex in an acidic environment.

### Possible anticancer mechanism of [Cu(L)(Ind)NO_3_] and the HSA complex

**Figure 7:**
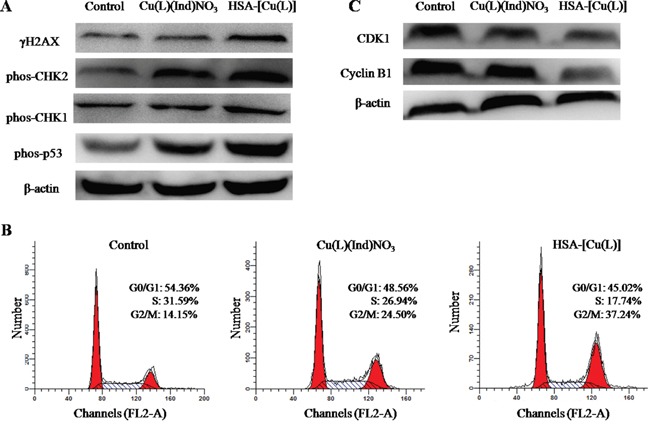
**A.** Immunoblotting analysis of proteins (γH2AX, phos-CHK1, phos-CHK2 and phos-p53) related to the DNA damage pathway. *β*-Actin was assessed as a loading control. **B.** The cell cycle distribution histograms of MCF-7 cells treated with [Cu(L)(Ind)NO_3_] and HSA-[Cu(L)] at the same concentration of 1.4 μM for 24 h. **C.** The expression levels of CDK1 and cyclin B1 in MCF-7 cells induced by [Cu(L)(Ind)NO_3_] and HSA-[Cu(L)] at the same concentration (1.4 μM), determined by Western blot analysis. Cells untreated are used as a control, and *β*-actin is the loading control. Each experiment group is repeated three times.

Most previously outlined metal compounds are assumed to encourage cell death by damaging cell DNA [58, 59]. We therefore investigated the expression of biomarkers associated with that DNA damage pathway. MCF-7 cells were incubated with the [Cu(L)(Ind)NO_3_] compound and the HSA complex (HSA-[Cu(L)]) at an identical concentration for 24 hours. Untreated MCF-7 cells were used as a negative control. The Western blot data show that the phosphorylated forms of CHK1, CHK2, H2AX (γH2AX) and p53 (Ser15) proteins expression are up−regulated slightly by [Cu(L)(Ind)NO_3_] compound in comparison with the control, whereas the expressions of the phosphorylated proteins are markedly enhanced by the HSA complex (Figure 7A). This result indicates that the HSA complex kill cells by damaging DNA [60–62].

Cell cycle arrest and apoptosis are two of the most common cellular response to DNA damage [63], and we therefore monitored features related to these pathways. As shown in Figure 7B, the present outcomes show that the [Cu(L)(Ind)NO_3_] compound and the HSA-[Cu(L)] cause an accumulation of cells in the G2/M phase of the cell growth cycle. Advancement through the cell cycle is tightly controlled by cyclin complexes and cyclin−dependent kinases (CDKs) at the different phases [64]. To continue investigating the mechanisms that took part in the HSA-[Cu(L)] anticancer actions, CDK1 and cyclin B1 were studied. Compared with the vehicle−treated control, the expression level of cyclin B1 was decreased by the [Cu(L)(Ind)NO_3_] compound and the HSA-[Cu(L)], especially for the HSA-[Cu(L)]. Similar outcomes were also noted for CDK1 after treatment with [Cu(L)(Ind)NO_3_] compound and the HSA-[Cu(L)] (Figure 7C).

**Figure 8:**
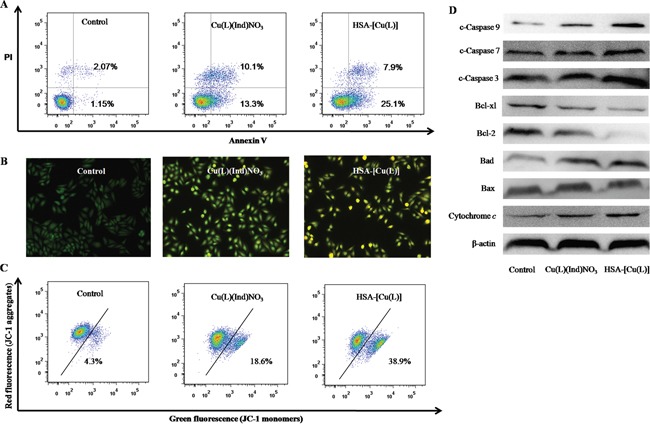
**A.** Representative dot plots of PI and Annexin V double staining on the MCF-7 cells in the presence of the indicated concentration (1.4 μM) of [Cu(L)(Ind)NO_3_] and HSA-[Cu(L)]. **B.** Representative images of AO/EB double stained MCF-7 cells after treatment with complexes [Cu(L)(Ind)NO_3_] and HSA-[Cu(L)] the indicated concentration (1.4 μM). **C.** Effects of [Cu(L)(Ind)NO_3_] and HSA-[Cu(L)] at the same concentration (1.4 μM) on mitochondrial membrane potential analyzed by JC-1 staining and flow cytometry. **D.** The expression levels of the Bcl-2 family proteins and the caspase family members in Bel-7402 cells induced by [Cu(L)(Ind)NO_3_] and HSA-[Cu(L)] at the same concentration (1.4 μM) for 24 h.

Many metal drugs generally exert their cytotoxic effects through apoptosis. In this study, the FITC-Annexin V/PI method was utilized to figure out whether the degradation of MCF-7 cells incubated with the HSA-[Cu(L)] was induced by apoptosis. Results from Annexin V-FITC/PI staining shows that the percentage of MCF-7 cell apoptosis is 23.4% for the [Cu(L)(Ind)NO_3_] compound and 33% for the HSA-[Cu(L)] (Figure 8A). Apoptotic cells usually demonstrate changes in cell morphology; e.g., blebbing, nuclear fragmentation and chromatin condensation. MCF-7 cells treated with [Cu(L)(Ind)NO_3_] compound display morphologic signs of apoptosis, whereas cells treated with the HSA-[Cu(L)] show distinct morphological changes that are characteristic of apoptosis (Figure 8B). Hence, both flow cytometry assay and morphological features provide validation for the apoptosis pathway.

The mitochondrial transmembrane potential (Δψ_m_) regulates the permeability of the mitochondrial pore, which plays a vital role in provoking apoptosis. The changing of mitochondria pore can be shown by several important events such as the decrease in mitochondrial membrane potential, the emergence of mitochondrial permeability transition, and the release of cytochrome *c* [65]. To determine whether or not apoptosis induced by [Cu(L)(Ind)NO_3_] compound and the HSA-[Cu(L)] is mediated by mitochondrial dysfunction, Δψ_m_ was examined by flow cytometry using JC-1. As shown in Figure 8C, treatment of MCF-7 cells with [Cu(L)(Ind)NO_3_] compound and the HSA-[Cu(L)] causes a reduction in Δψ_m_ to varying amounts, confirming the provocation of mitochondria-mediated apoptosis. Western blot analysis (Figure 8D) revealed that [Cu(L)(Ind)NO_3_] compound and the HSA-[Cu(L)] inhibited the expression of Bcl-2 and Bcl-xl (prosurvival Bcl-2 family proteins), and encouraged the expression of Bcl-2 associated death promoter, or BAD. The ratio of Bcl-xl/BAD and Bcl-2/BAX is reduced, suggesting that the Bcl-2 family of proteins regulates the loss of Δψ_m_. In addition, immunoblotting studies revealed that cytochrome *c* and cleaved caspase family proteins (cleaved caspase 3, 7 and 9) were enhanced. These data strongly suggest that the HSA-[Cu(L)] can effectively trigger mitochondria-mediated apoptosis by regulating the expression of Bcl-2 family proteins compared with [Cu(L)(Ind)NO_3_].

## DISCUSSION

To enhance the *in vitro* specificity, anticancer and delivery efficiency of Cu-containing agent, we could design Cu pro-drugs modeled on the structure and behavior of the HSA IIA subdomain. Indeed, our results show that it is feasible to develop [Cu(L)(Ind)NO_3_] as a pro-drug by using the special by-products (Lys199 and His242) of the HSA IIA subdomain because the HSA complex structure showed that Lys199 and His242 replaced indazole and NO_3_^−^ of [Cu(L)(Ind)NO_3_] and coordinated with Cu(II). This reaction allows the Cu compound to bind well to the hydrophobic cavity of the HSA IIA subdomain. Additionally, only a limited amount of Cu compound (approximately 5%) was released from the HSA complex at pH 7.4. In contrast, the binding affinity of [Cu(L)(Ind)NO_3_] to the HSA carrier drastically decreased in an acidic environment, and the amount of Cu compound (approximately 80%) released from the HSA complex. These results suggested that the HSA complex would be stable in the blood during *in vivo* circulation and that the Cu compound would be released after selective accumulation into the acidic lysosomes of cancer cells.

*In vivo* therapeutic efficiency of the HSA complex was evaluated by considering 3 aspects, namely, selectivity, associated side effects, and TIR. The TIR of the HSA complex reached 64.6% and was approximately 1.8-times higher than that of [Cu(L)(Ind)NO_3_] (35.2%). Mice treated with the HSA complex showed no significant reduction in their body weight, while those treated with [Cu(L)(Ind)NO_3_] showed a significant weight loss. H&E staining indicated that damage to the liver and kidneys was greatly reduced in mice treated with the HSA complex in comparision with that in mice treated with [Cu(L)(Ind)NO_3_]. Importantly, results of ICP-AES showed that the HSA complex facilitated the accumulation of the Cu compound into tumors *in vivo*. Taken together, the results of breast cancer xenograft experiments in mice indicated that the HSA complex was associated with lesser side effects and better antitumor activity and resulted in the selective accumulation of the Cu compound in tumor cells compared with the free Cu compound.

**Figure 9:**
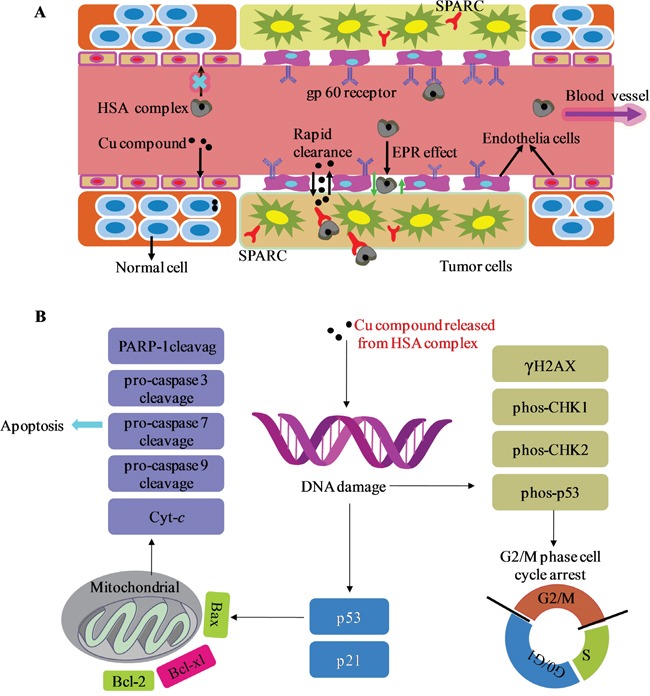
**A.** General schematics of the delivery process of HSA complex through normal and cancerous tissues. **B.** Proposed cellular mechanism of action. *Abbreviations*: SPARC, secreted protein acidic and rich in cysteine; EPR effect, the enhanced permeability and retention effect.

Why does the HSA complex showed stronger *in vivo* selectivity toward tumor cells compared with the Cu compound alone. It is attributable to the unique characteristics of the tumor tissue and the HSA carrier (Figure 9A) [28]. Unlike blood vessels in most normal tissues, ongoing angiogenesis in tumor tissues produces vascular gaps (≥200 nm) between adjacent endothelial cells [66–68]. HSA (diameter, approximately 15 nm) accumulates in the leaky vasculature surrounding tumor cells by the enhanced permeability and retention (EPR) effect and therefore is delivered more preferentially to tumor cells [28]. In contrast, the [Cu(L)(Ind)NO_3_] compound penetrates both tumor and normal cells, but the [Cu(L)(Ind)NO_3_] agent, as a small molecule, was shown to be rapidly cleared from the tumor interstitium [69, 70]. This unique property of the HSA carrier supports naked Cu compound to decrease cytotoxicity against normal cells and enhance cytotoxicity against cancer cells. In addition, the tumor endothelium expresses 2 albumin-binding proteins, namely, secreted protein acidic and rich in cysteine (SPARC) and gp60 receptor [71–73], which may facilitate the uptake and retention of the HSA complex in the tumor interstitium. Subsequently, HSA complex enters into the tumor cells by endocytosis [74]. The Cu compound released from the HSA complex then eliminates cancer cells, possibly through diverse anticancer mechanisms (Figure 9B) that induce DNA damage (Figure 7A), resulting in activation of the p53 pathway, cell cycle arrest at the G2/M phase (Figure 7B), and mitochondria-mediated apoptosis by regulation of the expression of Bcl-2 family proteins (Figure 8).

In conclusion, owing to Lys199 and His242 of HSA replacing with leaving groups of Cu pro-drug and coordinating with Cu^2+^, the delivery efficiency and anticancer activity of Cu compound has been improved through tighter binding to the IIA subdomain of HSA, and by selectively accumulating in tumor tissue. Compared to the Cu compound alone, the HSA complex showed better tolerance, higher drug accumulation in tumor tissues, and lower toxicity, indicating that it had superior antitumor activity and was associated with lesser side effects. These results suggested that the pro-drug strategy with an HSA carrier for the i.v. administration of novel, active aroylhydrazone Schiff base-containing Cu(II) pro-drugs may be a novel and effective approach for targeted cancer therapy.

## MATERIALS AND METHODS

Human serum albumin (fatty acid content < 0.05%, catalogue number A3782) was bought from the Sigma Chemical Company and used without additional purification. All other chemicals and solvents utilized were undiluted and accessible at commercial sources. Elemental analyses (C, N and H) of all complexes were performed on a PerkinElmer series II CHNS/O 2400 analyzer. Infrared (IR) spectra were recorded using KBr pellets (4000−400 cm^−1^) on a Nexus 870 FT-IR spectrophotometer. UV−visible spectra were measured at room temperature using a Cary 1E UV−visible spectrophotometer in the 200−800 nm range.

### Synthesis and characterization of [Cu(L)(Ind)NO_3_]

(*E*)-*N'*-(5-bromo-2-hydroxybenzylidene)benzohydrazide Schiff base ligand (HL) was synthesized according to previous reported methods [52].

Cu(NO_3_)_2_·3H_2_O (0.48 g, 2 mmol) was dissolved in 15 mL of methanol. To this, a solution of HL ligand (0.64 g, 2 mmol) and indazole (0.23 g, 2 mmol) in methanol (15 mL) was added. The combined solutions were stirred at room temperature for 24 hours to result in a celadon solution and then filtered. The filtrate was kept uncovered for a week, and formed blue block crystals. The crystals were isolated, washed three times with distilled water and dried in a vacuum desiccator containing anhydrous CaCl_2_. Yield: 820 mg (73%). *Anal*. Calcd for C_21_H_16_BrCuN_5_O_5_ (561.84): C, 44.79; H, 2.87 and N, 12.46. Found: C, 44.93; H, 2.89 and N, 12.27. IR (KBr, cm^−1^): 1618 í(C=N); 567, 514, 459, 445 í(Cu−N/Cu−O).

### Structure determination of Cu(II) compound

A Bruker APEX CCD X-ray diffractometer controlled by the *APEX2* software was utilized to collect the diffraction of graphite-monochromated Mo−Kα (*λ* = 0.71073 Å) radiation from the crystal at room temperature. Empirical adsorption corrections were applied to all data using SADABS. The structures were solved by direct methods and improved against *F*^2^ by full-matrix least-squares methods using the SHELXTL version 5.1 [75]. All of the non-hydrogen atoms were improved anisotropically. Hydrogen atoms were placed at calculated positions and restricted to ride on their parent atoms. Crystallographic data for compound [Cu(L)(Ind)NO_3_] data are provided in Supplementary Tables S1 and S2. Crystallographic data for the structural analyses have been deposited at the Cambridge Crystallographic Data Centre, reference number 1418472.

### X-ray crystallography of HSA complex

Palmitic acid (PA) was dissolved in alcohol and diluted to 2.5 mM by 20 mM pH 7.5 potassium phosphate. Fatty acid (FA) free HSA was purified by removing HSA dimers and multimers as published [76]. The complexes of Cu(II) compounds and HSA were prepared by mixing 100 μL HSA (100 mg/mL), 380 μL PA (2.5 mM) and 90 μL Cu compound (5 mM) overnight. The mixture was then concentrated to 100 mg/ml with a Millipore spin filter (10000 Da cutoff). Crystallization was performed by setting drop vapor diffusion at room temperature. An equal volume of the HSA complex was mixed with the reservoir solution, consisting of 28−32% (w/v) polyethylene glycol 3350, 50 mM potassium phosphate (pH 7.5), 5% glycerol, and 4% DMSO. Crystals were directly selected from the drop solution and then frozen in liquid nitrogen.

X-ray diffraction data were collected under cryo-conditions (100 K) using the Shanghai Synchrotron Radiation Facility. The data were integrated and scaled with HKL2000. The data set obtained from the HSA complex was processed in space group *P*1. The structure of the HSA complex was solved by molecular replacement with the AMORE program using the HSA-MYR structure (PDB code 1BJ5) stripped of its ligands as the model. The model was initially enhanced using a rigid body protocol in CNS and then subjected to cycles of positional and B-factor refinement before the calculation of the initial 2*F*_o_ − *F*_c_ and *F*_o_ − *F*_c_ maps was set. These maps were used to guide the position of the fatty acid and drug ligands and to make manual changes to the protein before performing more cycles of refinement. Figures representing the structure were prepared by PyMOL [77]. Data unit cell parameters and collection details are given in Table 3.

**Table 3:** Data collection statistics and crystallographic analysis of HSA complex

Data collection	
Space group	*P*1
Cell parameters, *a, b, c* (Å)	95.46, 95.64, 38.50
Cell parameters,	104.82, 90.05, 101.86
Resolution range (Å)	27−2.3
Data redundancy	4.1
Completeness (%)^a^	98% (98.7%)
*I*/σ	14.1 (4.4)
*R*_merge_ (%)^b^	7.5% (24.4%)
Model refinement	
*R*_model_ (%)^c^	24.68%
*R*_free_ (%)^d^	29.96%
r.m.s. deviation from ideal bond lengths	0.008 Å
r.m.s. deviation from ideal angles (°)	1.175

a Values for the outermost resolution shell are given in parentheses.

b *R*_merge_=100×Σ_h_Σ_j_| I_hj_-I_h_|/Σ_h_Σ_j_ I_hj_ where I_h_ is the weighted mean intensity of the symmetry-related refractions I_hj_.

c *R*_model_=100×Σ_hkl_|F_obs_-F_calc_|/Σ_hkl_F_obs_ where F_obs_ and F_calc_ are the observed and calculated structure factors, respectively.

d *R*_free_ is the *R*_model_ calculated using a randomly selected 5% sample of reflection data omitted from the refinement.

### Cell culture

MCF-7 human breast cancer cell lines, MCF-7/ADR drug-resistant cells, and WI-38 normal lung fibroblast cells (purchased from the American Type Culture Collection and the German Collection of Microorganisms and Cell Cultures) were cultured in Culture medium DMEM or RPMI 1640. The culture mediums contain 10% fetal bovine serum (FBS), 50 U/mL of penicillin, and 50 mg/mL of streptomycin at 37°C under a humidified atmosphere containing 5% CO_2_.

### Cytotoxicity assay (MTT)

The colorimetric MTT assay was used to determine the toxicity of the compound [Cu(L)(Ind)NO_3_] and its HSA complex. One hundred microliters of cell suspension at a density of 5 × 10^4^ cells per mL was seeded in 96-well plates and incubated for 24 hours at 37°C in 5% CO_2_. Then the medium was replaced with a replacement medium comprised of 10% FBS containing the compounds at various concentrations and incubated at 37°C under conditions of 5% CO_2_. The final concentration of DMSO in each well was 0.5% and this amount was present in the untreated control as well. After 48 hours, 20 μL of 5 mg/mL MTT assay stock solution in PBS was added to each well, and the plate was incubated for another 4 hours. Then, the obtained blue formazan crystals were dissolved in 200 μL well^−1^ DMSO. The absorbance was read by an enzyme labeling instrument (Infinite M2000) with a 570/630 nm double wavelength measurement. The cytotoxicity was measured byte percentage of cell survival compared with the negative control. The final IC_50_ values were calculated by the Bliss method (*n* = 5). Each test was repeated in at least three independent experiments.

### *In vivo* animal studies

All animal experiments were performed in compliance with the Animal Management Rules of the Ministry of Health of the People's Republic of China (document NO. 55, 2001) and the guidelines for the Care and Use of the University of Jinan Ethics Committee. Female NOD/SCID mice were obtained from Beijing HFK Bioscience Co., Ltd.

### Acute toxicity study

The acute toxicity of the [Cu(L)(Ind)NO_3_] and HSA complex were assessed on normal mice as method described previously [78]. Briefly, 24 healthy Kunming mice (aged 3∼4 weeks, weighed 18∼22 g) were split into three groups, with 8 mice in each group. Free Cu compound and HSA complex were given to the different groups of mice at a dose of 13 μmol Cu/kg body weight (i.v.). Saline (NaCl) was administered to the control group. Blood samples from each group of mice were drawn 3 days post-injection to prepare the serum samples. At this juncture the serum biochemical parameters of alanine aminotransferase (ALT), aspartate aminotransferase (AST), blood urea nitrogen (BUN) and creatinine kinase (CK) were determined. Finally, the heart, liver and kidney were sectioned for histopathological analysis with hematoxylin and eosin (H&E) staining.

### *In vivo* antitumor activity study

The female nude mice were injected subcutaneously in the right flank region with 200 μL of cell suspension containing 5 × 10^6^ MCF-7 cells. *β*-Estradiol cypionate (3 mg/kg) was administered i.m. every 7 days. When the tumour volume was approximately 100 mm^3^ for anti-tumour activity study, the mice were randomly divided into 3 groups, 8 animals in each group. The MCF-7 tumor-bearing mice were intravenously injected with NaCl, free Cu compound and HSA complex at dose of 2.5 μmol Cu/kg body weight every 3 days. Each mouse of different group was earmarked and followed individually throughout the whole experiments. The width and length of the tumor and the body weight of mice were measured before every injection by the end of experiment. The volume was calculated based on the following equation: tumor volume (V) = 1/2 × length × width^2^. After 24 days of treatment, mice were euthanized and tumor tissues were excised for histopathological analysis with H&E staining and TUNEL assay.

### Selective of HSA complex *in vivo*

At the end of the experiment *in vivo*, the tumors of mice were homogenized, placed in teflon containers and mineralized in a microwave apparatus under pressure (system Milestone MSL 1200) in the presence of 1 mL of 30% hydrogen peroxide and in 7 mL of concentrated HNO_3_. The contents of Cu in mice tumor and major organs were determined using inductively coupled plasma atomic emission spectrometry (ICP-AES).

### Cu pro-drug release from HSA complex

The Cu(II) compound release from HSA complex was studied by dialyzing HSA complex at pH 4.7 and 7.4 buffers (citric-phosphate buffers) to simulate cell matrix and interstitial space environment, respectively [56, 57]. Briefly, 2 mL HSA complex suspension in dialysis pocket were dispersed in tube containing 40 mL pH 4.7 and 7.4 buffers for 48 h, respectively. The amount of Cu(II) compound released from the HSA complex was determined by graphite furnace atomic absorption spectrometer (GF-AAS).

### Apoptosis by flow cytometry

The assay was carried out according to the manufacturer's directions for the Annexin V-FITC Apoptosis Detection Kit (Abcam). Briefly, MCF-7 cells seeded into 6-well plates were exposed to the tested compounds at the indicated concentrations for 12 hours. MCF-7 cells without the treatment were used as a control. The cells were then harvested and re-suspended in 500 μL annexin-binding buffer. Next, the cell suspension was stained with 5 μL annexin V and 5 μL PI at room temperature with no light source for 15 minutes, and then analyzed immediately by flow cytometry (FACScan, Bection Dickinson, San Jose, CA).

### Acridine orange/ethidium bromide (AO/EB) double staining

MCF-7 cells were treated with [Cu(L)(Ind)NO_3_] compound (1.4 μM) and HSA complex (1.4 μM) for 12 h, and then washed once with ice-cold PBS and fixed with 4% paraformalclehyde. Cells were washed with ice-cold PBS twice, stained with a medium containing AO/EB solution (100 μg/mL AO, 100 μg/mL EB) in the dark for 10 min. After washing the cells twice with PBS, the cells with observed morphological change were obtained under a reflected fluorescence microscope (Nikon MF30 LED, Japan).

### Cell cycle distribution analysis

Cell cycle distribution was analyzed by flow cytometry and PI staining. Briefly, MCF-7 cells were treated with [Cu(L)(Ind)NO_3_] compound (1.4 μM) and HSA complex (1.4 μM). For cell cycle analysis, treated cells were collected, washed twice with ice-cold PBS and fixed with 70% ethanol at 4°C overnight. Next, cells were treated with Rnase A (100 μg/ml) for 30 minutes at 37°C, followed by PI staining for 30 minutes in the dark. The cell cycle was analyzed by flow cytometry (FACScan, Bection Dickinson, San Jose, CA).

### The change of mitochondrial membrane potential assay

JC-1 probe (Beyotime, Haimen, China) was employed to measure mitochondrial depolarization in MCF-7 cells. The cells were treated with the [Cu(L)(Ind)NO_3_] compound (1.4 μM) and HSA complex (1.4 μM) for 12 h and collected by centrifugation. The cells were resuspended and stained with 0.5 mL of JC-1 (10 μg/mL) stock solution for 30 min at 37°C in the dark. Subsequently, the fluorescence of separated cells was detected with a flow cytometer (FACScan, Bection Dickinson, San Jose, CA).

### Western blot analysis

MCF-7 cells were seeded in 3.5 cm dishes at a density of 5.0 × 10^5^ cells/well in 5 mL of complete DMEM and allowed to attach for 24 h at 37°C. The cells were treated with [Cu(L)(Ind)NO_3_] and HSA complex at indicated concentrations for 24 h. The cells untreated were used as a negative control. After treatment for 24 h, the cells were harvested and washed with ice-cold PBS three times, and then they were lysed in radioimmunoprecipitation assay (RIPA) buffer supplemented with inhibitors of proteases and inhibitor of proteases and inhibitor of phosphatases sodium orthovanadate. The protein concentration of the supernatant was determined by BCA (bicinchoninic acid) assay. Equal amounts of cellular total proteins were separated on 10% SDS−polyacrylamide gel electrophoresis and then transferred onto polyvinylidene difluoride membranes (Millipore, MA, USA) and blocked with 5% nonfat milk in TBST buffer (20 mM Tris, pH 8.0, 150 mM NaCl, and 0.05% Tween 20) for 1 h. Then, the membranes were incubated with the β-actin, CDK1, Cyclin B, γH2AX, phos-CHK2, phos-CHK1, phos-p53, cleaved caspase-9, cleaved caspase-7, cleaved caspase-3, Bcl-xl, Bcl-2, Bad, Bax and cytochrome *c* primary antibodies (Cell Signaling Technology and Sigma) overnight at 4°C. After a subsequent washing step, the membrane is incubated with the appropriate anti-mouse or anti-rabbit secondary antibodies (Cell Signalling Technology) conjugated with horseradish peroxidase for 1 h at room temperature and washed for three times with TBST. The immunoreactivity was detected using Amersham ECL Plus (Amersham) western blotting detection reagents.

### Statistical analysis

The experiments were repeated three to five times and results were expressed as the mean ± standard deviation (SD). Student's *t* test was applied to evaluate the significance of differences measured, and the differences between groups with *p* < 0.05 were considered to be significant.

## SUPPLEMENTARY MATERIAL FIGURES AND TABLES


