# Supplementary material for: Developing an anticancer copper(II) pro-drug based on the nature of cancer cell and human serum albumin carrier IIA subdomain: mouse model of breast cancer
Source: Oncotarget. 2016 Aug 22;7(41):67004–19. doi: 10.18632/oncotarget.11465 (PMC5341853; doi:10.18632/oncotarget.11465)
Supplement: Supplementary file 1 [file oncotarget-07-67004-s001.pdf]

Developing an anticancer copper(II) pro-drug based on the nature of cancer cell and human serum albumin carrier IIA subdomain: mouse model of breast cancer

SUPPLEMENTARY MATERIALS

The interactions between Cu compound and HSA by spectrum analysis

Fluorescence spectrum

HSA solution (1 µM) was titrated by successive additions of the Cu(II) compound using micropipettes for all of the experiments. The fluorescence emission spectra were scanned from 300 to 420 nm after excitation at 280 nm (the maximum emission was obtained at 347). The binding constant of HSA for compound can be analyzed according to the equation [1]:

$$\log[(F_0 - F)/F] = \log K + n \times \log(Q)$$

where F and F_0 are the fluorescence intensities of protein in the presence and absence of the quencher, respectively; n is the number of binding sites; K is the binding constant and [Q] is quencher concentration. From the plot of $\log[(F_0 - F)/F]$ versus log [Q], the number of binding sites (n) and the binding constant (K) were calculated.

UV−visible spectrum

UV−visible absorption spectra were measured on a Cary 1E UV−Visible spectrophotometer in the 200−800 nm range, connected to a Haake F3 water bath, which maintained the temperature of each sample at 37°C. Measurements were performed by using 30 µM HSA and 30 µM solutions of each sample in 1 mL volumes.

Matrix-assisted laser desorption ionization time-of-flight mass spectrometry (MALDI-TOF-MS) analyses

HSA protein solution (1.5 mM) was added to solutions of the Cu(II) compound in order to achieve a 4:1 metal/protein ratio and shaken for 24 h at room temperature. The samples were prepared using the dried droplet method with freshly prepared sinapinic acid [10 mg/mL in CH_3CN/H_2O/trifluoroacetic acid (70:29.9:0.1)] as the matrix solution. The protein sample solution (0.1 mL, series of 1:10 dilutions) was mixed on the target with the matrix solution (0.1 mL) and allowed to air-dry. The MS spectra were recorded in the *m/z* 30000−100000 range in a positive linear mode.

REFERENCE

1. Abou-Zied OK, Al-Shihi OI. Characterization of subdomain IIA binding site of human serum albumin in its native, unfolded, and refolded states using small molecular probes. J Am Chem Soc. 2008; 130: 10793-10801.

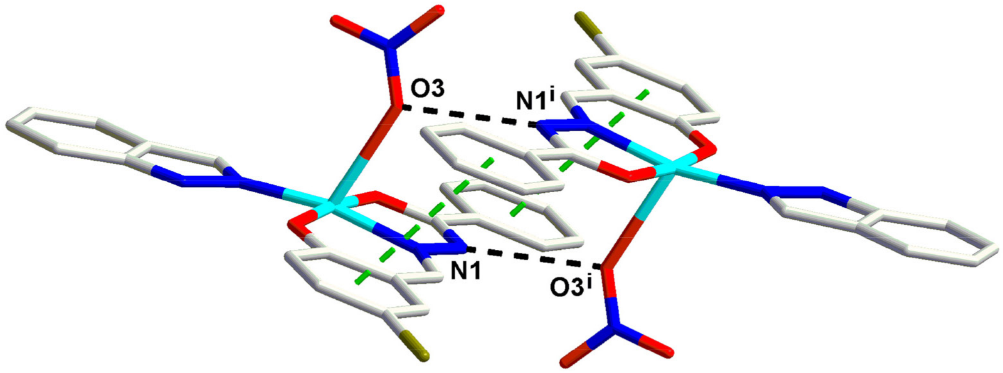

Supplementary Figure S1: The N–H···O interactions (black dash lines) and π···π interactions (green dash lines) in Cu(L)(Ind)NO_3 (Symmetry code: i = 1 − *x*, − *y*, 2 − z).

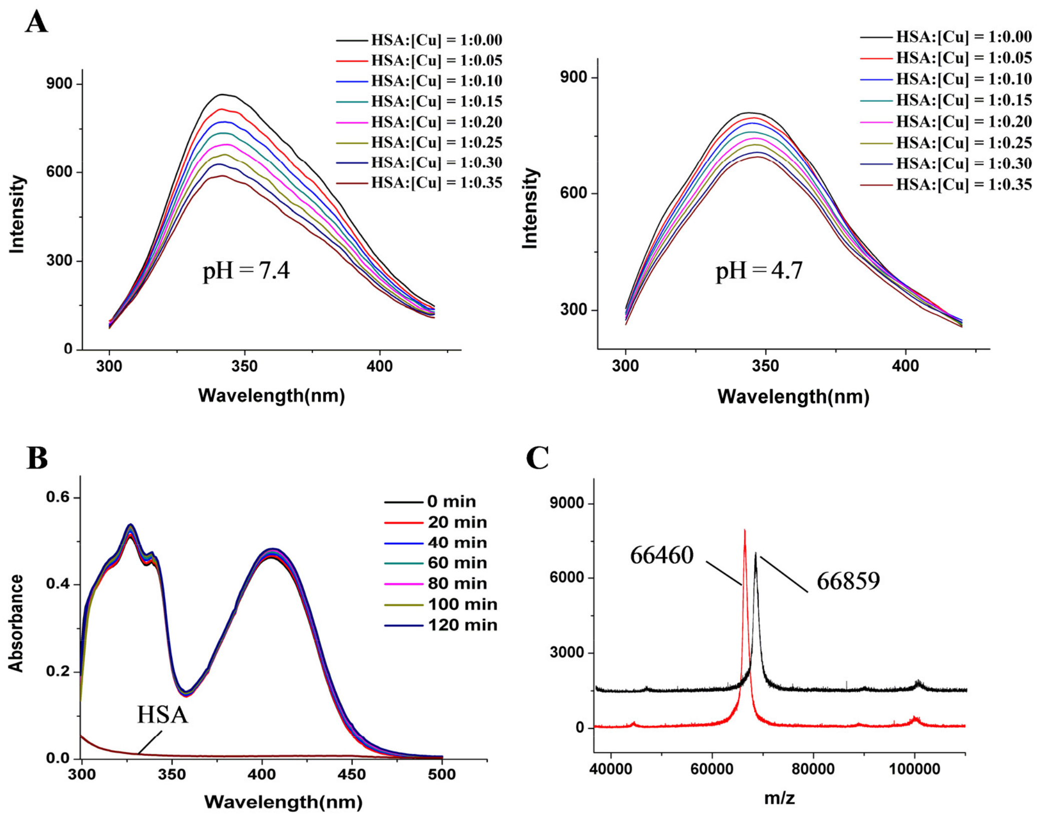

Supplementary Figure S2: A. Fluorescence quenching spectra of HSA by different concentrations of [Cu(L)(Ind)NO_3], T = 298 K. **B.** UV−Vis spectra for [Cu(L)(Ind)NO_3] (30 μM) incubated with HSA in PBS solution, 0.5% DMSO at 37°C for 2 h. **C.** MALDI-TOF-MS spectra of HSA-[Cu(L)(Ind)NO_3] conjugate.

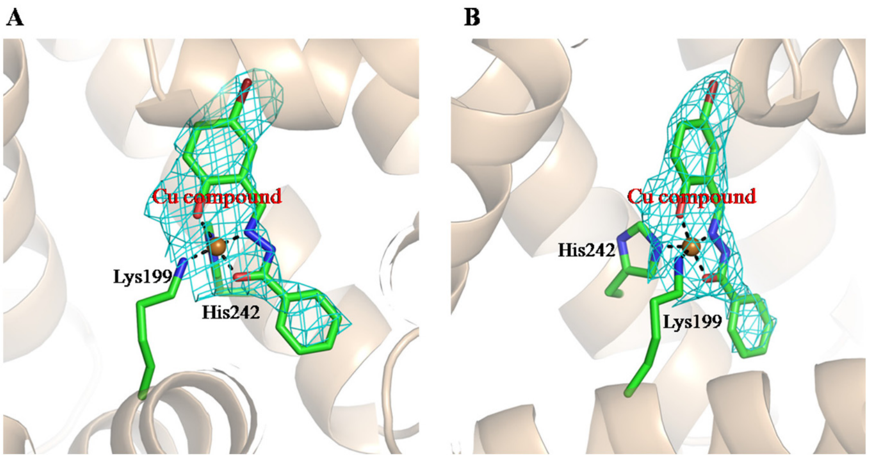

Supplementary Figure S3: The experimental σA weighted Fo − Fc electron density map (2.5 σ) of [Cu(L)(Ind)NO_3] in HSA from different angels.

Supplementary Table S1: Crystal data for compound [Cu(L)(Ind)NO_3]

Compound	Cu(L)(Ind)NO_3
Empirical formula	$C_{21}H_{16}BrCuN_5O_5$
Molecular weight	561.84
Crystal system	triclinic
Space group	*P*–1
a (Å)	8.1499(19)
b (Å)	10.614(2)
c (Å)	14.071(3)
α (°)	97.467(3)
β (°)	104.716(3)
γ (°)	110.415(3)
T (K)	296.15
V (Å^3)	1070.7(4)
Z	2
$\rho_{calc.}$ (g·cm^{-3})	1.743
F(000)	562
μ(Mo-K$_\alpha$) (mm^{-1})	2.931
Data/restraint/parameters	4378/0/298
Goodness-of-fit on F^2	1.079
Final R_1, wR_2 [$I > 2\sigma(I)$]	0.0401, 0.1073

Supplementary Table S2: Selected bond lengths [Å] and angles [º] in compound [Cu(L)(Ind)NO_3]

**Cu(L)(Ind)NO_3**			
Cu1−O1	1.879(2)	O1−Cu1−N2	93.49(11)
Cu1−N2	1.919(2)	O1−Cu1−O2	173.88(10)
Cu1−O2	1.963(2)	O1−Cu1−N3	90.42(11)
Cu1−N3	1.966(3)	N2−Cu1−O2	81.76(11)
Cu1−O3	2.474(3)	N2−Cu1−N3	166.12(12)
O2−Cu1−N3	93.33(11)		

Supplementary Table S3: IC_{50}[a] (μM) values of Cu(II) compound and its HSA complex toward a panel of cancer cell lines for 48 h

compound	Antitumor activity IC_{50} (μM)		
	**SK-N-MC**	**DMS-53**	**SK-Mel-28**
[Cu(L)(Ind)NO_3]	2.18 ± 0.22	3.38 ± 0.29	1.96 ± 0.23
HSA-[Cu(L)]	1.69 ± 0.12	1.82 ± 0.17	1.35 ± 0.14
*Cis*platin	13.64 ± 1.29	9.76 ± 1.28	18.37 ± 1.63

[a] IC_{50} values are presented as the mean ± SD from three separated experiments.
